# Implementing an Educational Module for Orthopaedic Residents Placing Skeletal Traction

**DOI:** 10.5435/JAAOSGlobal-D-22-00165

**Published:** 2023-04-14

**Authors:** Ena Nielsen, Zakkary Walterscheid, Daphne Beingessner, Conor Kleweno

**Affiliations:** From the Department of Orthopaedic Surgery and Sports Medicine, Harborview Medical Center, Seattle, WA.

## Abstract

Although placement of a distal femoral traction (DFT) pin is a relatively simple procedure used to stabilize femoral and pelvic fractures, it places patients at risk of iatrogenic vascular, muscular, or bony injury. We designed and implemented an educational module combining theory and practical experience to standardize and improve resident teaching on the placement of DFT pins.

**Methods:**

We introduced a DFT pin teaching module into our second-year resident “boot camp,” which is used to help prepare residents for taking primary call in the emergency department at our level I trauma center. Nine residents participated. The teaching module included a written pretest, an oral lecture, a video demonstration of the procedure, and a practice simulation on 3D printed models. After completing the teaching, each resident underwent a written examination and proctored live simulation involving 3D models using the same equipment available in our emergency department. Pre-teaching and post-teaching surveys were used to assess resident experience and confidence with placing traction in the emergency department.

**Results:**

Before the teaching session, the rising postgraduate year 2 residents scored an average of 62.2% (range, 50% to 77.8%) on the DFT pin knowledge quiz. This improved to an average of 86.6% (range, 68.1% to 100%) (*P* = 0.0001) after the teaching session. After completing the educational module, they also demonstrated an improvement in confidence with the procedure, from 6.7 (range, 5 to 9) to 8.8 (range, 8 to 10) (*P* = 0.04).

**Discussion:**

Despite reporting high levels of confidence in their ability to place traction pins before starting the postgraduate year 2 consult year, many residents also reported anxiety around the accurate placement of traction pins. Early results of our training program showed improved resident knowledge of safe placement of traction pins and improved confidence with the procedure.

Skeletal traction, whether intramedullary or extramedullary, has been used in the treatment of diaphyseal femur fractures since 1519 when Guy de Chauliac described suspending a weight attached to the leg over a pulley system at the end of the bed.^1^ Transcondylar pins were first described in 1907 by Steinmann.^2^ Although skeletal traction is not commonly used as definitive treatment in the modern setting, it is frequently used for fracture stabilization in the setting of femur, acetabular, and unstable pelvic fractures.

The distal femoral traction (DFT) pin allows for longitudinal traction up to 15% bodyweight and allows for axial fracture reduction and soft-tissue rest.^3,4^ When placed correctly, traction pins do not cause long-term knee dysfunction and can often be more comfortable for patients who underwent the application of a long leg splint.^3^ However, this procedure is not without several risks and potential complications. Good knowledge of the anatomy around the knee is needed to avoid iatrogenic knee capsule penetration, which may place patients at risk of septic arthritis, or damage to neurovascular structures.^5^ In addition, there have been case reports of complications such as pin site infection, iatrogenic fracture, sciatic nerve palsy, and heterotopic ossification associated with traction pins.^6-8^

At our institution, we place approximately 400 to 500 traction pins in the emergency department per year. This procedure is typically done by the postgraduate year (PGY)-2 resident on trauma consults. Training in this procedure is limited and highly variable. Most often PGY-1 residents learn from PGY-2 residents using the antiquated “see one-do one” model. Rarely, residents get the opportunity to place traction pins in the operating room under the supervision of an attending physician before beginning their trauma consult rotation.

This variability represents an educational opportunity with the potential to improve patient safety, the quality of patient experience in the emergency department, and resident experience. There is an abundance of evidence in the orthopaedic and general surgery literature supporting boot camps, surgery simulators, and low-cost surgical modules as methods for improving residents' manual skills before treating real patients.^9,10,11,12^ In an effort to better standardize the training for placement of DFT pins, we designed a low-cost simulation and educational module for our PGY-2 residents. We predicted that resident comfort with the procedure and knowledge of indications, techniques, and complications would be improved after implementation of this module.

## Methods

Before beginning this study, all PGY-1 through PGY-5 orthopaedic surgery residents at our institution were surveyed regarding their experiences with placing DFT pins at our level I trauma center. This included questions about conflicts with other providers, how they learned to do the procedure, and their level of confidence with the procedure. We also left a free response option for recommendations on how to improve the procedure. No residents were excluded from the study, and completion of the survey was voluntary.

Based on feedback from this survey, we developed a DFT pin teaching module for our PGY-2 boot camp. This boot camp is used to help prepare rising PGY-2 residents to take primary trauma call. Our module was multifaceted and included an initial written quiz to establish a baseline knowledge level, an oral lecture on indications for traction, important anatomic landmarks, how to properly place a traction pin, and risks of traction. Time was given after the lecture for a question-and-answer session with senior residents and a trauma attending. After the lecture, there was a narrated video demonstration of a DFT pin being placed. The residents were then allowed to practice the procedure using a low-fidelity simulation using both hand and power drills on 3D printed models.

During each PGY-2 resident’s first block of trauma call, they were required to retake the written quiz to assess their comprehension of the DFT procedure. In addition, they underwent a practical examination using a simulated scenario and 3D model proctored by an attending. After all PGY-2 residents had completed their first block of trauma call, the initial survey was readministered to evaluate their experience and confidence in placing DFT pins.

All surveys and quizzes were developed in REDCap electronic data capture tools hosted at our health system.^13,14^ Statistics were analyzed using Stata/IC 14.0.^15^ Paired Student *t*-test analysis was used to compare pre-training and post-training survey results. Descriptive statistics were used for demographic information.

## Results

67.5% (n = 27/40) of residents responded to the initial survey, including 77.8% (n = 7/9) of the rising PGY-2 residents. 100% of the rising PGY-2 residents completed the remainder of the quizzes and surveys. A summary of resident concerns about the process of placing DFT pins and ideas for improvement is provided in Table 1. Three main concerns arose regarding traction pins: ability to place them accurately (51.9%, n = 14/27), ability to provide adequate pain control (33.3%, n = 9/27), and equipment availability or failures (33.3%, n = 9/27). This information guided our educational process as noted earlier in the methods section.

**Table 1:** Preliminary Resident Survey Results Regarding the Placement of Distal Femoral Traction Pins

Survey	% (n = 27)
What aspects of placing traction pins were/are you most worried about?	
Pain	51.9 (14)
Equipment availability or failure	33.3 (9)
Accurate placement	33.3 (9)
How do you think we could improve the placement of traction pins at our institution?	
Power drills	92.6 (25)
Education	33.3 (9)
Increase supplies	29.6 (8)
Unknown	3.7 (1)

Before the teaching session, the nine rising PGY-2 residents scored an average of 62.2% (range, 50% to 77.8%) on the DFT pin knowledge quiz. This improved to an average of 86.6% (range, 68.1% to 100%) (*P* = 0.0001) after the teaching session.

**Figure 1:**
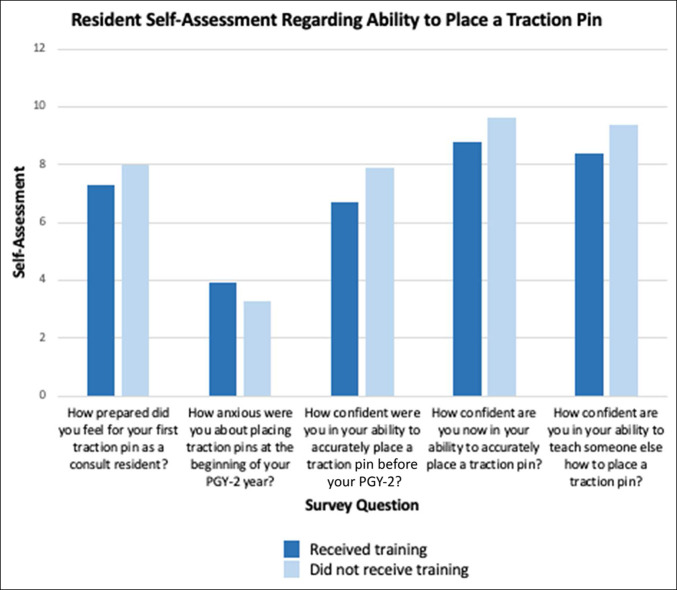
Resident self-assessment regarding ability to place a traction pin. PGY = postgraduate year

After completing the PGY-2 consult year, senior residents' confidence in their ability to place a traction pin improved from 7.5 (range, 3 to 10) to 9.6 (range, 7 to 10), and they felt confident in their ability to teach junior residents how to properly place a traction pin (9.4; range, 5 to 10) (Table 3; Figure 1). After completing the educational module and a single 6-week trauma rotation, the current PGY-2 class also demonstrated an improvement in confidence with the procedure, from 6.7 (range, 5 to 9) to 8.8 (range, 8 to 10) (*P* = 0.04). However, they demonstrated no difference in anxiety with placing pins before and after teaching (3.9; range, 2 to 5 versus 3.9; range, 1 to 7; *P* = 0.95) (Table 2).

**Table 2:** Resident Self-assessment Regarding Ability to Place a Distal Femoral Traction Pin

Survey	Before Teaching (n = 7)	After Teaching (n = 9)	*P* Value
How anxious are you about placing traction pins?	3.9 (range, 1-7)	3.9 (range, 0-9)	0.95
How confident are you in your ability to accurately place a traction?	6.7 (range, 4-8)	8.8 (range, 8-10)	0.04^a^

a Statistically significant.

All assessments were on a 0 to 10 scale, with zero being the least and 10 being the most.

PGY-2 residents who received the teaching session and more senior residents who did not rated their anxiety before placing their first traction pin similarly (3.9; range, 1 to 7 versus 3.3; range, 0 to 9; *P* = 0.41). They also felt similarly prepared for the procedure before beginning their first consult block (7.3; range, 5 to 10 versus 8.0; range, 0 to 10; *P* = 0.52) (Table 3; Figure 1).

**Table 3:** Comparison of Resident Self-assessment Regarding Ability to Place a Distal Femoral Traction Pin Between PGY-2 Residents Who Received the Education Module and Senior Residents Who Did Not

Survey	PGY-2 Residents (n = 9)	Senior Residents (n = 18)	*P* Value
How prepared did you feel for your first traction pin placement as a consult resident?	7.3 (range, 5-10)	8.0 (range, 0-10)	0.52
How anxious were you about placing traction pins at the beginning of your PGY-2 year?	3.9 (range, 1-7)	3.3 (range, 0-9)	0.41
How confident were you in your ability to accurately place a traction pin before your PGY-2?	6.7 (range, 4-8)	7.9 (range, 3-10)	0.11
How confident are you now in your ability to accurately place a traction pin?	8.8 (range, 8-10)	9.6 (range, 7-10)	0.04^a^
How confident are you in your ability to teach someone else how to place a traction pin?	8.4 (range, 7-10)	9.4 (range, 5-10)	0.07

PGY = postgraduate year

a Statistically significant.

All assessments were on a 0 to 10 scale, with zero being the least and 10 being the most.

## Discussion

DFT pin placement is a common procedure at our institution. Despite this, historically residents have been asked to conduct the procedure without prior standardized training. We, therefore, designed and implemented an education module for our PGY-2 boot camp to improve resident experience with the procedure.

During the course of implementing this new module, we solicited informal feedback from the rising PGY-2 residents regarding their experiences and efficacy of the teaching. While this feedback was generally positive, this was not necessarily borne out in our surveys. Despite a demonstrable improvement in written knowledge about DFT pin placement, those who received the education and those who did not felt similarly prepared for the procedure and had similar levels of preprocedure anxiety and confidence in their abilities. This disparity may highlight a need for more intensive hands-on, practical training rather than written modules. While we thought it was important that the residents prove they understood the concepts of the procedure before actually conducting the procedure, it may be beneficial to increase the amount of time we spend on practical demonstrations in future modules.

Several limitations of this study may contribute to these findings. Because this is the first year this module was implemented, we do not have a large number of residents who participated in the teaching session to compare with prior years. This small data set means that outliers have a larger effect on the average data set. It is difficult to control for differences in resident personality, such as baseline confidence levels, anxiety, and accuracy of self-assessment, within this study. These differences combined with the small numbers likely contributed to our research findings. We plan to continue this module as a permanent part of our PGY-2 boot camp. As we repeat the surveys with subsequent groups of residents, these limitations and errors may decrease.

In addition, we are comparing real-time data from the rising PGY-2 residents with recalled data from the remainder of the residents in our program. This introduces a large amount of bias—multiple studies have shown that research participants tend to forget negative experiences related to the self and have overconfidence in past performance based on current performance. Time elapsed since the event also contributes to errors in recall.^16,17^ A resident in their PGY-5 will certainly rate their procedural confidence higher than a PGY-2 resident who has completed a single trauma rotation, making comparison of residents' current confidence and teaching abilities limited. While we considered selecting a group of residents to not receive the education and act as a control group, we thought the benefits of standardized training to patient care and resident safety were too great to justify this.

Confounding this further is the fact that the post-teaching surveys were administered after residents had completed a single 6-week block of trauma consults. This was due to difficulty coordinating resident schedules for conducting the teaching modules and delays in resident completion of surveys. Regardless, the changes in resident confidence and anxiety may be partially attributable to their real-world experience on trauma consults rather than entirely because of the teaching module.

This study primarily focused on resident self-assessment of their ability to conduct DFT pin placement and their written understanding of the technique. Although these are both important aspects to safely conduct procedures, we had no measured data on their actual technical skills. Residents were required to conduct traction pin placement safely and accurately on a 3D model, as judged by an attending physician, but this was simply pass/fail. Previous studies of surgical simulations have demonstrated improved skill after simulation. Ruder et al. conducted a study comparing drill plunging depth before and after the educational session. After the sessions, plunging depth decreased markedly, from a mean of 1.5 cm to a mean of 0.5. Similarly, Lopez et al demonstrated that after a 4-week training module, medical students were able to drastically improve their accuracy and speed in a variety of orthopaedic skills, including triangulation and plunge depth.^9,10,11,12,18^ In future iterations of this module, it may be worthwhile to implement a measure of technical competency beyond the pass/fail model.

Despite a reportedly high level of confidence in their ability to place traction pins before starting the PGY-2 consult year, many residents also reported anxiety around the accurate placement of traction pins and their ability to provide adequate pain control to patients. Suggestions for process improvement included improving resident education. Early results of our training program showed improved resident knowledge of safe placement of traction pins. We are continuing to implement quality improvement initiatives to increase resident and patient safety with this procedure.
